# Short-Term Touch-Screen Video Game Playing Improves the Inhibition Ability

**DOI:** 10.3390/ijerph18136884

**Published:** 2021-06-26

**Authors:** Boyu Qiu, Yanrong Chen, Xu He, Ting Liu, Sixian Wang, Wei Zhang

**Affiliations:** 1 Key Laboratory of Brain, Cognition and Education Sciences, South China Normal University, Ministry of Education, Guangzhou 510631, China; boyuqiu@m.scnu.edu.cn (B.Q.); 2020023430@m.scnu.edu.cn (Y.C.); 2019023008@m.scnu.edu.cn (X.H.); tingliu@m.scnu.edu.cn (T.L.); 2020023449@m.scnu.edu.cn (S.W.); 2 School of Psychology, South China Normal University, Guangzhou 510631, China; 3 Center for Studies of Psychological Application and Guangdong Key Laboratory of Mental Health and Cognitive Science, South China Normal University, Guangzhou 510631, China

**Keywords:** executive function, video game, shifting, updating, inhibition, common executive function

## Abstract

There is mixed evidence regarding whether video games affect executive function. The inconsistent results in this area may have to do with researchers’ conceptualizations of executive function as a unified construct or as a set of independent skills. In the current study, 120 university students were randomly assigned to play a video game or to watch a screen record of the video game. They then completed a series of behavioral tasks to assess the shifting, updating and inhibiting subcomponents of executive function. Scores on these tasks were also used as indicators of a component-general latent variable. Results based on analysis of covariance showed that, as predicted, the inhibition subcomponent, but not the updating or the shifting subcomponent, was significantly enhanced after gaming. The component-general executive function was not enhanced after gaming once the results were controlled for other subcomponents. The results were unrelated to participants’ self-reported positive and negative affect. The findings add key evidence to the literature on executive function and potentially contribute to the therapeutic use of video games to maintain executive function in the aged population.

## 1. Introduction

Touchscreen video games are now part of the daily lives of many people, a large number of whom play on smartphones or tablets. The number of video game players worldwide reached 2.60 billion in 2020, with average weekly gameplay hours ranging from 6.69 to 7.98 across countries [1]. Studies on cognitive training have tested whether there are cognitive benefits associated with video gaming. Evidence of cognitive enhancement is robust when referring to visual attention [2,3] but is mixed when referring to higher cognitive functions, such as action inhibition and set-shifting [4]. These latter skills are part of executive function (EF), the ability that controls and regulates goal-directed behavior [5]. The current study examines whether the mixed results with regard to the benefits of gaming on EF are related to how EF is conceptualized and measured.

Many studies have shown a positive correlation between video gaming and EF. Long-term training-based studies [6,7,8,9] have supported significant improvements in participants’ performance on EF tasks after a period of video-game training. For example, Sosa and Lagana [10] reported that 15 h of video-game training over five weeks improved EF as measured by a card-sorting task and a Stroop task. In studies with short-term video-game playing under laboratory conditions and subsequent measurement of EF [11,12], video games were also found to increase EF. For example, Buelow, et al. [13] found that short-term video-game play improved college students’ EF as measured by the Iowa Gambling Task, the Balloon Analogue Risk Task and the Wisconsin Card Sorting Task. The idea that video games can enhance EF is also supported by studies comparing high-frequency and low-frequency video-game players [14,15,16,17,18]. For example, Al-Gabbani, et al. [19] reported that children (mean age 9.2 years) with experience playing action video games outperformed children who did not have experience, on a spatial span task and a stop-signal task. A meta-analysis from Ong et al. [20] concluded that video games could significantly improve EF.

However, other studies have found only partial benefits or negative effects of video-game playing on EF. For long-term video-game training, Ruiz-Marquez et al. [21] reported that 15 video-game training sessions of 30 min each did not have a significant positive effect on EF measured by the Stroop task. For short-term video-game playing, Parong and Mayer [22] found that participants who played virtual-reality games for a short period did not outperform the control group on the n-back task. Hummer et al. [23] found that short-term violent-video-game exposure did not significantly influence reaction time on a go-nogo task. For studies comparing individuals with different game frequencies, Huang et al. [24] showed that, compared to non-video-game players, frequent-video-game players did not perform better on inhibition tasks. A meta-analysis of 118 studies on the effects of video gaming [25] showed that only the inhibition component of EF was significantly enhanced after gaming. There was no effect on other EF skills like multitasking, switching, and working memory. The effect of video gaming on EF was not supported in reviews for studies with children [26] or elder samples [27].

The conflicting results regarding the effects of video-game playing on EF may be due in part to whether EF is conceptualized as a unitary construct or as a set of independent skills. Wang et al. [3] defined EF as an entity, an umbrella term for planning, working memory, reasoning, inhibition, mental flexibility and the monitoring of action. Using this definition of EF, the researchers found no link between video-game playing and EF. Eggenberger et al. [28] conceptualized EF as a general construct with three subcomponents, shifting, inhibition and working-memory updating. They found a positive effect of video gaming only on the inhibition subcomponent. Therefore, it is possible that playing video games influences some, but not all, aspects of EF. Wang et al.’s [3] meta-analysis might have mixed the effects from different components and come to a different conclusion than Eggenberger et al.’s [28]. Nevertheless, it is also possible that research participants only demonstrate the strategies or response patterns required for a specific task or component of EF, making it difficult to demonstrate improvement in EF across tasks or components. Unlike these earlier studies, we argue that EF is both an entity and subdivided and that the component-general and the component-specific factors of EF should be studied simultaneously.

In early attempts to subdivide EF, Miyake et al. [29] proposed three subcomponents, namely inhibition (inhibit prepotent responses), shifting (shift between task demands) and updating (update and operate in working memory), which can be measured by separate tasks. Confirmatory factor analysis provided support for this model of EF. Friedman et al. [30] built on this model by proposing a common EF latent variable that links all the tasks from the three subcomponents. Statistical tests only partially support this conceptual model. Specifically, after adding the common EF factor as a latent variable, the inhibition subcomponent could not be extracted from the model (i.e., a large part of its variance was explained by the common EF factor) and thus, was excluded. This result was the basis of a new bifactor model of EF [30]. The bifactor model assumes that the structure of EF is both unitary (a common EF factor can predict all task performance) and specific (different subcomponents of EF may have different effects across different tasks). This model provides a framework for studying the component-general and the component-specific factors simultaneously.

The common factor and inhibition shared a large amount of variance in Friedman et al.’s [30] study. Both general EF (component-general) and inhibition (a component-specific aspect of EF) may be improved after gaming. A meta-analytic study [25] showed that only the inhibition subcomponent was enhanced after video-game training. [28] found enhanced inhibition but not shifting and updating. Using an antisaccade task as a measure of the inhibition component, Diarra et al. [31] found improvement in oculomotor inhibition in older adults trained on the *Super Mario* video game. However, in general these studies assessed the subcomponents of EF without attention to an overall EF construct. One exception was Eggenberger et al. [28], who used the Montreal cognitive assessment to measure the common EF factor, in addition to the specific components of inhibition, shifting and updating. But the results of this study are difficult to interpret because the common EF factor was measured using a single task instead of estimating from task performance across the three subcomponents. It is unclear whether this measure of common EF actually represents a domain-general ability across EF tasks.

In sum, the current study tested both component-general and component-specific EF factors in healthy young adults who were randomly assigned to play a touchscreen video game or watch a screen record of the video game. A touchscreen video game similar to *Pac-Man* was used for this study. On the one hand, most video-game players in China play through their touchscreen phones [32]. On the other hand, social information displayed in the video game may affect participants’ attentional processing [33]. The type of game used in the current study conveys less social information.

We hypothesized that: Compared to the control group that watched the video, the video-game group would (1) perform better on the post-game measure of the inhibition ability, (2) not perform better on the post-game measure of the updating ability and (3) not perform better on the post-game measure of the shifting ability. No hypothesis about common EF was made because there is no similar evidence for a common EF score extracted from the three EF subcomponent measures.

## 2. Method

### Participants

One hundred and twenty undergraduate students (average age: 21.29 years, *SD*: 2.47 years) were recruited to participate in the study; 45% of the participants were women (average age: 21.00 years, *SD*: 2.83 years), and 55% were men (average age: 21.53 years, *SD*: 2.13 years). The participants were randomly assigned to the video-game group or the control group.

The materials and procedure of this study were approved by the Research Ethics Committee of the university with which the first author is affiliated. Written informed consent was obtained from each participant upon arrival. Participants were told that their performance scores were anonymous and that they had the right to withdraw at any time.

## 3. Instruments

### 3.1. Positive Affect and Negative Affect Schedule

The positive and negative affect schedule (PANAS) [34] was administered at the beginning of the study to measure the participants’ self-reported positive and negative affect. Participants rated 20 items using a 5-point Likert scale (1 = very slightly or not at all, 5 = extremely) on the extent to which they were experiencing positive and negative emotions. Internal consistency (measured by the Cronbach’s *α* coefficient) of the PNANS in the current study was 0.83 for the positive-affect subscale and 0.87 for the negative-affect subscale.

### 3.2. Video Game

**Figure 1:**
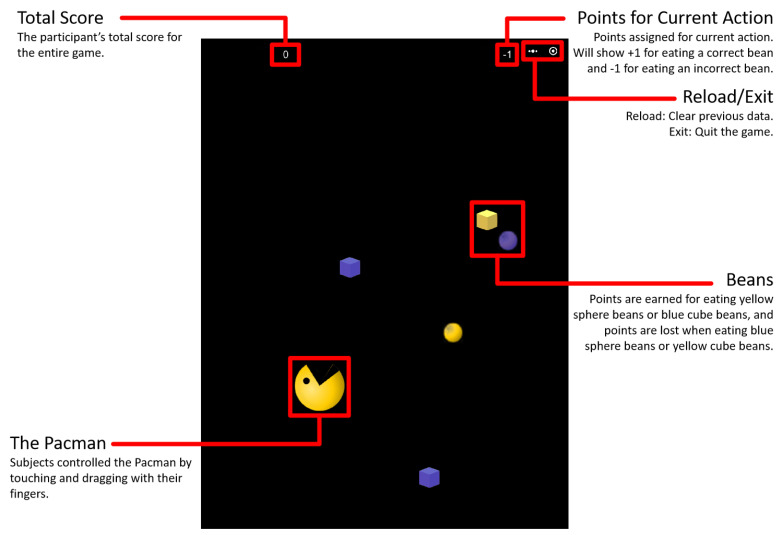
Interface of the touchscreen video game. *Note.* Screenshot of the video game interface on a 10.2-inch touchscreen. Red boxes and lines are not part of the interface.

The video game was similar to the game of *Pac-Man*. In the game used in our study, the participant guided a spherical creature with a large mouth to eat beans that fell from the top of the screen. Points were awarded when the creature ate “correct” beans and lost when the creature ate “incorrect” (distractor) beans. Correct beans showed a specific combination of shape and color. Sphere-shaped beans earned points when they were yellow but cost points when they were blue; cube-shaped beans earned points when they were blue but cost points when they were yellow. The video game was programmed using Java script and run on a 10.2-inch touchscreen. The interface is illustrated in Figure 1. The materials created for this study did not infringe on any copyrights. 

### 3.3. Post-Game Questionnaire

After they finished gaming, participants in the video game group rated their game experience [33]. Participants used a 10-point scale (0 = totally disagree, 9 = totally agree) to rate whether the game was action-packed, enjoyable, exciting, entertaining, fun, involving, hard to play and frustrating; the extent to which they felt able to play the game; and whether the game had violent or prosocial content. Example items were “The game involves helping behavior” and “The game is hard to play”.

### 3.4. Components of EF

After-game measures of the executive function components were based on earlier research [30]. Details about these tasks can also be found in Miyake et al. [29]. The tasks chosen for use in the current study were selected to cover as many modalities as possible (paper–pencil, auditory and visual tests) to avoid common method bias.

### 3.5. Plus-Minus Task

The plus–minus task aims to measure the shifting component of EF. The materials are three lists of numbers, each presented on a separate page. Each list contains 30 randomly generated two-digit numbers arranged in a row. On the first list, participants were asked to add three to each number; on the second list, to subtract three from each number; and on the third list, to alternate between adding three and subtracting three in sequence (i.e., add three to the first number, subtract three from the second number, add three to the third number, and so on). The participants wrote down their answer to each item as they progressed through each list. The cost of shifting between plus and minus (as an indicator of shifting ability) was calculated as the time cost of the third list (measured by a stopwatch) minus the average time cost of the first and second lists. The indicator of shifting ability is the inverse of shifting cost, so that larger numbers indicate better executive function.

### 3.6. Tone-Monitoring Task

The tone-monitoring task measures the updating component of EF. Participants were first given a short test to make sure they were able to identify different tones, including high-pitched tones (880 Hz), medium-pitched tones (440 Hz) and low-pitched tones (220 Hz). After the hearing test, participants practiced on 25 tones to familiarize themselves with the rules of the task. In the task itself, participants heard a series of 25 tones (a mixed order of 8 high-/medium-/low-pitched tones and a random tone) in each of four blocks. Each tone lasted 500 ms with an inter-stimulus interval of 2500 ms. Participants were asked to respond by pressing a button when every 4th tone of each particular pitch was presented. For example, in the sequence ”low, high, medium, high, high, low, low, *high*, high, *low*,” participants should respond to the 4th high tone and the 4th low tone (italicized). When an incorrect button was pressed, the tone count for that pitch automatically reset to zero, and feedback was presented on the screen. Note that the tone count was not shown on the screen, which means that participants had to monitor the number of times each pitch had been presented. The number of correct button presses (possible range: 0–24) was used as the indicator of updating.

### 3.7. Antisaccade Task

The antisaccade task measures the inhibition component of EF. In each trial of the task, a fixation point (a cross presented in the center of the screen) was first presented in the center of the screen for a variable amount of time (randomly selected from 1500–3500 ms in 250 ms intervals), followed by a visual cue (a solid black square) on one side of the screen (left or right) for 225 ms. The target stimulus (an arrow in a hollow square, pointing either up or down) was then presented on the opposite side of the cue for 150 ms. Participants were asked to indicate the direction of the arrow by pressing the corresponding button, which required them to inhibit their attention to the cue stimulus in order to identify the direction of the target. Participants practiced on 22 trials before they received 90 task trials. The average accuracy across all task trials was used as the indicator of inhibition. Note that “antisaccade” refers to the name of the task and does not mean using the eye-tracking technique.

### 3.8. The Common EF

Based on the bifactor model [35], latent variable modeling was used to extract the common EF from the three task indicators. The loading of the common EF factor was estimated based on the covariance matrix using maximum likelihood estimation, and the indicator of the common EF was calculated as the sum of the three weighted standardized task indicators (with each weight assigned based on the corresponding standardized path coefficient). The model was estimated using Mplus Version 7.0 (Muthén & Muthén, Los Angeles, CA, USA).

## 4. Procedure

Participants were randomly assigned to the video-game group or the control group upon arrival, and then they completed the PANAS. After that, participants in the video-game group were given a brief tutorial on playing the game. They practiced for 5 min and played the game for 30 min. They then completed the post-game questionnaire. Participants from the control group watched a screen record of the video game for 35 min instead of actually playing the game. After that, all participants were given instructions about the EF tasks. They completed the plus–minus task, the tone-monitoring task and the antisaccade task in random order (balanced between subjects).

## 5. Data Analysis

To assess whether emotional state interfered with the results, we first conducted independent-sample *t* tests to examine whether the experimental and control groups differed in positive or negative affect at the beginning of the experiment. Pearson’s correlation coefficients between the PANAS scores and the EF task scores were also calculated to test whether positive or negative affect at the beginning of the experiment influenced task performance. To assess whether features of the game influenced performance on the EF tasks, the correlations between ratings from the post-game questionnaire and the task-performance scores were calculated within the experimental group. After examining the effect of these possible confounds, we conducted a series of analysis of covariances (ANCOVAs), investigated whether video-game exposure influenced one subcomponent of EF after controlled for the other subcomponents (e.g., controlled for inhibition and updating when analyzing between-group differences in shifting). Considering that the inhibition ability and the common EF shared a similar structure [30], the between-group differences of the common EF were tested by ANCOVA, controlled for inhibition. All analyses were performed using JASP software.

## 6. Results

### 6.1. Positive and Negative Affect as Possible Confounds 

The experimental and control groups did not differ in positive (*t* [118] = 1.015, *p* = 0.312, Cohen’s *d* = 0.185) or negative (*t* [118] = 1.171, *p* = 0.244, Cohen’s *d* = 0.214) PANAS scores at the beginning of the experiment. Neither PANAS score was significantly related to any of the EF task indicators, *r*s ≤ 0.136, *p*s ≥ 0.140. Within the experimental group, no significant correlations emerged between ratings from the post-game questionnaire and EF task scores, *r*s ≤ 0.218, *p*s ≥ 0.094.

### 6.2. Influence of Video Gaming on Components of EF

The correlation matrix underlying the latent variable modeling was presented in Table 1. In latent variable modeling, standardized path coefficients from the common EF to shifting (*β* = 0.660, *p* < 0.001), inhibition (*β* = 0.666, *p* < 0.001) and updating (*β* = 0.559, *p* < 0.001) reached statistical significance. Descriptive statistics for the scores of inhibition, shifting, updating and the common EF are shown in Table 2, separately for each group. To present the scores of the three components on a common scale, the descriptive statistics for z-transferred scores of the three components are presented in Table 3, separately for each group. The results based on ANOVA and ANCOVA are presented in Table 4.

In support of our hypothesis, significant between-group differences were observed for the (1) inhibition component (F[1,116] = 4.684, *p* = 0.032, *η*^2^ = 0.033) but (2) not the shifting component (F[1,116] = 0.125, *p* = 0.725, *η*^2^ < 0.001) or (3) the updating component (F[1,116] = 0.009, *p* = 0.925, *η*^2^ < 0.001). (4) The between-group differences of the common EF (F[1,117] = 0.051, *p* = 0.822, *η*^2^ < 0.001) did not reach significant levels.

## 7. Discussion

The present study found that inhibition ability could be improved after short-term video games, consistent with previous results [28,31]. As a complement to and advancement of previous studies, we also examined the common EF extracted from three subcomponents after video gameplay. Soveri et al. [36] have argued that an actual improvement in cognitive training should include a broad transfer effect to different kinds of measures. Our results accommodate this argument by showing that inhibition ability was enhanced after gaming. In the present study, common EF did not show significant between-group differences when controlling for inhibition ability. However, this difference reached a significant level without controlling for the inhibition ability, *F*(1, 118) = 4.985, *p* = 0.027, *η*^2^ = 0.041. The common EF in this study was extracted from three measures. It may be controversial whether inhibition should be controlled when analyzing the common EF. The more tightly controlled result was selected for this study, and no significant group differences in common EF were reported.

A touchscreen game was used instead of a computer game in this study because the mobile phone with a touchscreen interface is currently the primary carrier of video games. In China, there were 640 million video game players in 2019, 620 million of whom were mobile-phone gamers [32]. Worldwide, video-game players who use a mobile phone (37%) or tablet (9%) outnumber those using personal computers (24%) [1]. The effects of video games based on mobile phones and tablets that use touchscreens may differ significantly from computer games that use keyboards. Testing the most recent and most common format seems most appropriate for the purposes of this study. Video games were run on personal computers in most previous studies in this area due to the technology available at the time. Whether these two carriers of video games (personal computers and touchscreen phones) would interact with the effect of video games on cognitive outcomes is worth further investigation.

As video games, especially mobile games, are enjoyable and easy to access, various populations may be motivated to play them [37]. Briefly playing a video game, as shown in this study, benefited the specific skill of inhibition. Playing touchscreen video games may maintain or improve EF ability and potentially contribute to other cognitive functions. In recent years, there has also been a growing body of research on the use of games to assess and train cognitive abilities in a variety of ways [38,39,40,41,42]. Specialized and standardized therapeutic applications of video gaming may help people with EF deficits to maintain cognitive functions [43].

## 8. Conclusions

The current study examined component-general and component-specific EF factors simultaneously after young adults played a brief touchscreen video game. The results showed that video game playing significantly improved inhibition ability. The results suggest the potential therapeutic applications of video games.

## Figures and Tables

**Table 1:** Correlation matrix underlying the latent variable modeling.

	Shifting	Updating	Inhibition
Shifting	1		
Updating	0.372 ***	1	
Inhibition	0.369 ***	0.439 ***	1

*Note. N* = 120. The shifting, updating and inhibition scores are measured by the plus–minus task (time difference in seconds), the tone-monitoring task (number of correct responses) and the antisaccade task (accuracy), respectively. *** *p* < 0.001.

**Table 2:** Means and standard deviations for the scores of executive function.

Scores	Video-Game Group (*N* = 60)		Control Group (*N* = 60)	
	*M*	*SD*	*M*	*SD*
Shifting	−15.34	13.05	−18.88	13.74
Updating	18.92	4.09	18.20	4.13
Inhibition	0.96	0.04	0.94	0.04
Common EF	0.29	1.40	−0.29	1.47

*Note.* The shifting, updating and inhibition scores were measured by the plus–minus task (time difference in seconds), the tone-monitoring task (number of correct responses) and the antisaccade task (accuracy), respectively. A larger number indicates better executive function. The common EF was a latent variable extracted from the three task indicators.

**Table 3:** Means and standard deviations for the Z-scores of the executive function components.

Scores	Video-Game Group (*N* = 60)		Control Group (*N* = 60)	
	*M*	*SD*	*M*	*SD*
Z (Shifting)	0.131	0.968	−0.131	1.022
Z (Updating)	0.087	0.996	−0.087	1.005
Z (Inhibition)	0.236	0.891	−0.236	1.054

*Note.* The shifting, updating and inhibition scores were measured by the plus–minus task (time difference in seconds), the tone-monitoring task (number of correct responses) and the antisaccade task (accuracy), respectively. A larger number indicates better executive function. The common EF was a latent variable extracted from the three task indicators.

**Table 4:** Results of ANOVA and ANCOVA.

Dependent	Type	Covariates		Degrees of Freedom		*F*	*p*	*η* ^2^
				Group	Residuals			
Shifting	ANCOVA	Updating	Inhibition	1	116	0.125	0.725	<0.001
Updating	ANCOVA	Shifting	Inhibition	1	116	0.009	0.925	<0.001
Inhibition	ANCOVA	Shifting	Updating	1	116	4.684	0.032	0.033
Common EF	ANCOVA	Inhibition		1	117	0.051	0.822	<0.001

*Note.* The shifting, updating and inhibition scores were measured by the plus–minus task (time difference in seconds), the tone-monitoring task (number of correct responses) and the antisaccade task (accuracy), respectively. The common EF was a latent variable extracted from the three task indicators. Levene’s test for equality of variances was performed before all analyses, and the results supported the homogeneity of the variance, *p*s > 0.138.

## Data Availability

The data and analysis codes used in this study are openly available in osf.io/rupfv at doi: 10.17605/OSF.IO/RUPFV. All materials for the study are available by email to the corresponding author.

## References

[1] Number of Gamers Worldwide 2020: Demographics, Statistics, and Predictions. https://financesonline.com/number-of-gamers-worldwide/.

[2] Bediou B., Adams D., Mayer R., Tipton E., Green C., Bavelier D. (2018). Meta-analysis of action video game impact on perceptual, attentional, and cognitive skills. Psychol. Bull..

[3] Wang P., Liu H., Zhu X., Meng T., Li H., Zuo X. (2016). Action video game training for healthy adults: A meta-analytic study. Front. Psychol..

[4] Kühn S., Gallinat J., Mascherek A. (2019). Effects of computer gaming on cognition, brain structure, and function: A critical reflection on existing literature. Dialogues Clin. Neurosci..

[5] Miyake A., Friedman N.P. (2012). The nature and organization of individual differences in executive functions. Curr. Dir. Psychol. Sci..

[6] Bertoni S., Franceschini S., Puccio G., Mancarella M., Gori S., Facoetti A. (2021). Action Video Games Enhance Attentional Control and Phonological Decoding in Children with Developmental Dyslexia. Brain Sci..

[7] Maillot P., Perrot A., Hartley A. (2012). Effects of interactive physical-activity video-game training on physical and cognitive function in older adults. Psychol. Aging.

[8] Stern Y., Blumen H.M., Rich L.W., Richards A., Herzberg G., Gopher D. (2011). Space fortress game training and executive control in older adults: A pilot intervention. Aging Neuropsychol. Cogn..

[9] Yu R., Leung G., Woo J. (2021). Randomized Controlled Trial on the Effects of a Combined Intervention of Computerized Cognitive Training Preceded by Physical Exercise for Improving Frailty Status and Cognitive Function in Older Adults. Int. J. Environ. Res. Public Health.

[10] Sosa G.W., Lagana L. (2019). The effects of video game training on the cognitive functioning of older adults: A community-based randomized controlled trial. Arch. Gerontol. Geriatr..

[11] McCord A., Cocks B., Barreiros A., Bizo L. (2020). Short video game play improves executive function in the oldest old living in residential care. Comput. Hum. Behav..

[12] Parong J., Wells A., Mayer R.E. (2020). Replicated evidence towards a cognitive theory of game-based training. J. Educ. Psychol..

[13] Buelow M.T., Okdie B.M., Cooper A.B. (2015). The influence of video games on executive functions in college students. Comput. Hum. Behav..

[14] Boot W.R., Kramer A.F., Simons D.J., Fabiani M., Gratton G. (2008). The effects of video game playing on attention, memory, and executive control. Acta Psychol..

[15] Li X., Huang L., Li B., Wang H., Han C. (2020). Time for a true display of skill: Top players in League of Legends have better executive control. Acta Psychol..

[16] Özçetin M., Gümüştaş F., Çağ Y., Gökbay İ.Z., Özmel A. (2019). The relationships between video game experience and cognitive abilities in adolescents. Neuropsychiatr. Dis. Treat..

[17] Palaus M., Viejo-Sobera R., Redolar-Ripoll D., Marrón E.M. (2020). Cognitive Enhancement via Neuromodulation and Video Games: Synergistic Effects?. Front. Hum. Neurosci..

[18] Yang X., Wang Z., Qiu X., Zhu L. (2020). The Relation between Electronic Game Play and Executive Function among Preschoolers. J. Child Fam. Stud..

[19] Al-Gabbani M., Morgan G., Eyre J.A. Positive relationship between duration of action video game play and visuospatial executive function in children. Proceedings of the 2014 IEEE 3rd International Conference on Serious Games and Applications for Health.

[20] Ong D., Weibin M.Z., Vallabhajosyula R. (2021). Serious games as rehabilitation tools in neurological conditions: A comprehensive review. Technol. Health Care.

[21] Ruiz-Marquez E., Prieto A., Mayas J., Toril P., Reales J.M., Ballesteros S. (2019). Effects of Nonaction Videogames on Attention and Memory in Young Adults. Games Health J..

[22] Parong J., Mayer R.E. (2020). Cognitive consequences of playing brain-training games in immersive virtual reality. Appl. Cogn. Psychol..

[23] Hummer T.A., Wang Y., Kronenberger W.G., Mosier K.M., Kalnin A.J., Dunn D.W., Mathews V.P. (2010). Short-term violent video game play by adolescents alters prefrontal activity during cognitive inhibition. Media Psychol..

[24] Huang V., Young M., Fiocco A.J. (2017). The Association Between Video Game Play and Cognitive Function: Does Gaming Platform Matter?. Cyberpsychol. Behav. Soc. Netw..

[25] Powers K.L., Brooks P.J., Aldrich N.J., Palladino M.A., Alfieri L. (2013). Effects of video-game play on information processing: A meta-analytic investigation. Psychon. Bull. Rev..

[26] Vedechkina M., Borgonovi F. (2021). A Review of Evidence on the Role of Digital Technology in Shaping Attention and Cognitive Control in Children. Front. Psychol..

[27] Gates N.J., Rutjes A.W., Di Nisio M., Karim S., Chong L.Y., March E., Martínez G., Vernooij R.W. (2019). Computerised cognitive training for maintaining cognitive function in cognitively healthy people in midlife. Cochrane Database Syst. Rev..

[28] Eggenberger P., Wolf M., Schumann M., de Bruin E.D. (2016). Exergame and balance training modulate prefrontal brain activity during walking and enhance executive function in older adults. Front. Aging Neurosci..

[29] Miyake A., Friedman N.P., Emerson M.J., Witzki A.H., Howerter A., Wager T.D. (2000). The unity and diversity of executive functions and their contributions to complex “frontal lobe” tasks: A latent variable analysis. Cogn. Psychol..

[30] Friedman N.P., Miyake A., Young S.E., DeFries J.C., Corley R.P., Hewitt J.K. (2008). Individual differences in executive functions are almost entirely genetic in origin. J. Exp. Psychol. Gen..

[31] Diarra M., Zendel B.R., Benady-Chorney J., Blanchette C.A., Lepore F., Peretz I., Belleville S., West G.L. (2019). Playing Super Mario increases oculomotor inhibition and frontal eye field grey matter in older adults. Exp. Brain Res..

[32] China Game Industry Report 2019. http://www.cgigc.com.cn/gamedata/21649.html.

[33] Qiu B., Zhen S., Zhou C., Hu J., Zhang W. (2020). Short-Term Prosocial Video Game Exposure Influences Attentional Bias Toward Prosocial Stimuli. Cyberpsychol. Behav. Soc. Netw..

[34] Watson D., Clark L.A., Tellegen A. (1988). Development and validation of brief measures of positive and negative affect: The PANAS scales. J. Personal. Soc. Psychol..

[35] Friedman N.P., Miyake A. (2017). Unity and diversity of executive functions: Individual differences as a window on cognitive structure. Cortex.

[36] Soveri A., Antfolk J., Karlsson L., Salo B., Laine M. (2017). Working memory training revisited: A multi-level meta-analysis of n-back training studies. Psychon. Bull. Rev..

[37] Ferguson C.J., Nielsen R.K., Maguire R. (2017). Do older adults hate video games until they play them?. A proof-of-concept study. Curr. Psychol..

[38] Bove R.M., Rush G., Zhao C., Rowles W., Garcha P., Morrissey J., Schembri A., Alailima T., Langdon D., Possin K. (2019). A Videogame-based digital therapeutic to improve processing speed in people with multiple sclerosis: A feasibility Study. Neurol. Ther..

[39] Aliah F., Ahmad I., Roszali F., Sarudin N. (2020). A review on mobile game learning applications trends. Int. J. Eng. Trends Technol..

[40] Song H., Yi D., Park H. (2020). Validation of a mobile game-based assessment of cognitive control among children and adolescents. PLoS ONE.

[41] Crepaldi M., Colombo V., Mottura S., Baldassini D., Sacco M., Cancer A., Antonietti A. (2020). The use of a serious game to assess inhibition mechanisms in children. Front. Comput. Sci..

[42] Homer B.D., Ober T.M., Rose M.C., MacNamara A., Mayer R.E., Plass J.L. (2019). Speed versus accuracy: Implications of adolescents’ neurocognitive developments in a digital game to train executive functions. Mind Brain Educ..

[43] Talaei-Khoei A., Daniel J. (2018). How younger elderly realize usefulness of cognitive training video games to maintain their independent living. Int. J. Inf. Manag..

